# Draft Genome Sequences of Vibrio cholerae Non-O1, Non-O139 Isolates from Common Tern Chicks (*Sterna hirundo*) following a Mass Mortality Event

**DOI:** 10.1128/MRA.01053-20

**Published:** 2020-11-12

**Authors:** Eckhard Strauch, Claudia Jäckel, Jens Andre Hammerl, Veit Hennig, Nicole Roschanski, Insa Dammann

**Affiliations:** a German Federal Institute for Risk Assessment, Department of Biological Safety, Berlin, Germany; b Department of Biology, Institute of Zoology, Universität Hamburg, Hamburg, Germany; c Landeslabor Schleswig-Holstein, Geschäftsbereich 2 (Veterinärwesen), Neumünster, Germany; University of Southern California

## Abstract

Vibrio cholerae is an inhabitant of aquatic environments worldwide. Here, we report the draft genome sequences of eight *V. cholera* non-O1, non-O139 isolates that were recovered from the corpses of two seabird chicks (common terns) following a mass mortality event in a German breeding colony in 2019.

## ANNOUNCEMENT

The presence of Vibrio cholerae in seabirds has been well known for many years (1, 2). Migrating birds are regarded as vectors for long-distance transport of these bacteria (3). Usually, the bacteria are regarded as commensals, while diseases of birds caused by V. cholerae are rarely reported.

In July 2019, a high chick mortality rate was observed in a colony of common terns (*Sterna hirundo*) in the saltmarshes of Neufelderkoog (District Dithmarschen) in the River Elbe Estuary (53°53′37.0″N, 8°58′55.21″E) (4). There, the seabird brood of 1 year (∼1,500 chicks) died within 1 week shortly before they were able to fly. To determine the cause of death, necropsies and microbiological investigations were conducted on two chick corpses. Tissue samples were taken from inner organs, and the samples were cultivated at 37°C on Columbia sheep blood agar (Thermo Fisher Scientific, Berlin, Germany) and MacConkey agar (Merck, Darmstadt, Germany) for 24 h and 48 h, respectively. Visible colonies were investigated using matrix-assisted laser desorption ionization–time of flight (MALDI-TOF) mass spectrometry (MALDI Biotyper; Bruker Daltonik, Bremen, Germany). V. cholerae non-O1, non-O139 isolates were recovered from liver, kidney, heart, small intestine, and lung, raising the possibility that these bacteria could be involved in the death of the birds. Herring as the sole feed for the chicks were suspected as a source of the infection. One V. cholerae isolate from each organ (eight isolates in total) (Table 1) was cultured on thiosulfate-citrate-bile-sucrose agar (Thermo Fisher Scientific) and ChromID *Vibrio* agar (bioMérieux, Marcy-l’Etoile, France) and sent to the Federal Institute for Risk Assessment.

**TABLE 1:** Phenotypic and genotypic features of the Vibrio cholerae isolates

Parameter	Data for isolate:							
	V917-19	V918-19	V919-19	V920-19	V921-19	V922-19	V923-19	V924-19
Isolation origin	Bird 1, lung	Bird 1, pericardium	Bird 1, gut	Bird 1, kidney	Bird 1, liver	Bird 2, kidney	Bird 2, liver	Bird 2, gut
Country of isolation	Germany	Germany	Germany	Germany	Germany	Germany	Germany	Germany
Yr of isolation	2019	2019	2019	2019	2019	2019	2019	2019
Phenotypic resistance	None	None	None	None	None	None	None	None
MIC (mg/liter)^*a*^								
Ampicillin	8	4	4	4	4	4	4	4
Azithromycin	≤2	≤2	≤2	≤2	≤2	≤2	≤2	≤2
Cefepime	0.25	0.12	0.12	0.12	0.12	0.12	0.12	0.12
Chloramphenicol	≤8	≤8	≤8	≤8	≤8	≤8	≤8	≤8
Ciprofloxacin	≤0.015	≤0.015	≤0.015	≤0.015	≤0.015	≤0.015	≤0.015	≤0.015
Colistin	>16	>16	>16	>16	>16	>16	>16	>16
Ertapenem	0.12	0.12	0.12	0.12	0.12	0.12	0.12	0.12
Cefotaxime	≤0.25	≤0.25	≤0.25	≤0.25	≤0.25	≤0.25	≤0.25	≤0.25
Cefoxitin	8	4	4	4	8	8	8	8
Gentamicin	2	1	1	2	1	2	2	1
Imipenem	2	2	2	2	2	2	2	2
Meropenem	0.25	0.25	0.25	0.25	0.25	0.25	0.25	0.25
Nalidixic acid	≤4	≤4	≤4	≤4	≤4	≤4	≤4	≤4
Sulfamethoxazole	≤8	≤8	≤8	≤8	≤8	≤8	≤8	≤8
Cefotaxime-clavulanic acid	≤0.06	≤0.06	≤0.06	≤0.06	≤0.06	≤0.06	≤0.06	≤0.06
Ceftazidime	≤0.25	≤0.25	≤0.25	≤0.25	≤0.25	≤0.25	≤0.25	≤0.25
Ceftazidime-clavulanic acid	0.25	≤0.12	≤0.12	≤0.12	≤0.12	≤0.12	≤0.12	≤0.12
Temocillin	4	2	2	2	2	2	2	2
Tetracycline	≤2	≤2	≤2	≤2	≤2	≤2	≤2	≤2
Tigecycline	≤0.25	≤0.25	≤0.25	0.25	≤0.25	≤0.25	0.25	≤0.25
Trimethoprim	≤0.25	≤0.25	≤0.25	≤0.25	≤0.25	≤0.25	≤0.25	≤0.25
Sequencing parameters								
No. of reads (total)	1,483,180	1,727,876	2,045,808	1,406,760	1,002,364	1,206,780	1,483,552	1,206,780
Average read length (bp)	270	274	275	275	276	273	274	273
No. of contigs	55	56	59	56	60	64	71	61
*N*_50_ (bp)	318,246	688,697	688,696	318,246	318,246	318,246	318,246	324,219
*L*_50_	4	3	3	4	4	4	4	4
Genome coverage (×)	25	28	30	25	20	20	23	20
SRA accession no.	SRR12520475	SRR12520474	SRR12520473	SRR12520472	SRR12520471	SRR12520470	SRR12520469	SRR12520468
Genomic features								
Genome size (bp)	4,089,403	4,076,756	4,089,668	4,088,449	4,089,048	4,088,105	4,089,034	4,089,730
GC content (%)	47.43	47.44	47.44	47.43	47.43	47.43	47.42	47.44
Total no. of genes	3,985	3,971	3,987	3,984	3,990	3,992	3,988	3,990
No. of coding genes	3,779	3,764	3,781	3,780	3,782	3,786	3,780	3,783
No. of CDSs^*b*^ (total)	3,872	3,857	3,874	3,872	3,877	3,880	3,874	3,876
No. of CDSs (coding)	3,779	3,764	3,781	3,780	3,782	3,786	3,780	3,783
Total no. of RNA genes	113	114	113	112	113	112	114	114
Total no. of rRNA genes (5S, 16S, 23S)	7, 7, 3	7, 7, 4	7, 7, 3	7, 7, 3	7, 7, 4	7, 6, 4	7, 7, 4	7, 7, 4
No. of complete rRNA genes	7, 1, 1	7, 1, 1	7, 1, 1	7, 1, 1	7, 1, 1	7, 0, 1	7, 1, 1	7, 1, 1
No. of partial rRNA genes	0, 6, 2	0, 6, 3	0, 6, 2	0, 6, 2	0, 6, 3	0, 6, 3	0, 6, 3	0, 6, 3
No. of tRNA genes	92	92	92	91	91	91	92	92
Total no. of pseudogenes	93	93	93	92	95	94	94	93
No. of predicted prophages^*c*^	2	1	2	2	2	2	2	2
47.7-kb K139 (GenBank accession no. NC_003313)	+	+	+	+	+	+	+	+
7.1-kb KSF-1phi (GenBank accession no. AY714348.1)	+	−	+	+	+	+	+	+
Plasmids^*d*^	ND	ND	ND	ND	ND	ND	ND	ND
Acquired antimicrobial resistance^*e*^	None	None	None	None	None	None	None	None
Sequence type^*f*^	Unknown	Unknown	Unknown	Unknown	Unknown	Unknown	Unknown	Unknown
BioProject no.	PRJNA563188	PRJNA563189	PRJNA563190	PRJNA563191	PRJNA563192	PRJNA563193	PRJNA563194	PRJNA563195
BioSample no.	SAMN12670120	SAMN12670121	SAMN12670122	SAMN12670123	SAMN12670124	SAMN12670125	SAMN12670126	SAMN12670133
GenBank accession no.	VTWK00000000.1	VTWL00000000	VTWM00000000.1	VTWN00000000.1	VTWO00000000.1	VTWP00000000.1	VTWQ00000000.1	VTWR00000000.1

a MICs were determined using broth microdilution according to the Clinical and Laboratory Standards Institute guidelines (13).

b CDSs, coding sequences.

c Analysis was conducted using PHASTER (https://phaster.ca) with default parameters. +, present; −, absent.

d Analysis was conducted using PlasmidFinder v2.1 (https://cge.cbs.dtu.dk/services/PlasmidFinder) with a 95% threshold for minimum identity and 60% minimum coverage. ND, not detected.

e Analysis was conducted using ResFinder v3.0 (https://cge.cbs.dtu.dk/services/ResFinder) with a 90% threshold for identity and 60% minimum length. The analysis of acquired determinants for the antimicrobial classes of aminoglycosides, β-lactams, colistin, fosfomycin, fusidic acid, macrolides, nitroimidazoles, oxazolidinones, phenicols, rifampin, sulfonamides, tetracyclines, trimethoprim, and glycopeptides yielded no matches.

f Analysis was conducted using MLST v2.0 (https://cge.cbs.dtu.dk/services/MLST) using the Vibrio cholerae scheme. All strains had identical alleles, as follows: *adk114*, 100% identity; *gyrB30*, 100% identity; *mdhE97*, 100% identity; *metE123*, 100% identity; *pntA66*, 100% identity; *purM9*, 100% identity; *pyrC* (novel allele), 99.78% identity to *pyrC147*.

For whole-genome sequencing, isolates were grown in lysogeny broth and genomic DNA was extracted with the PureLink genomic DNA kit (Invitrogen, Karlsruhe, Germany). MiSeq whole-genome sequencing (5) was conducted using the Nextera XT DNA sample preparation kit for library preparation and the MiSeq reagent 600-cycle v3 kit for paired-end sequence determination, as specified by the manufacturer (Illumina, Inc., San Diego, CA, USA). Raw reads were processed to quality-trimmed sequences using fastp v0.19.5 (https://github.com/OpenGene/fastp) with the following specifications: base limit, 50; length required, 15. Sequences were further checked with FastQC v1.0.4 (https://www.bioinformatics.babraham.ac.uk/projects/fastqc).

Automated *de novo* assembly (SPAdes v3.5.49) and genome annotation were performed using PATRIC (release 3.5.39) (6). Default parameters were routinely used for all software tools. Further information on software versions and parameters is given in Table 1. Bioinformatic analysis was conducted with the specified tools of the Center for Genomic Epidemiology (http://www.genomicepidemiology.org) and PGAP (National Center for Biotechnology Information) (7). Prophage prediction was performed with PHASTER (accessed 9 June 2019) (8).

Important phenotypic and genotypic features of the V. cholerae genomes are summarized in Table 1. Determination of antimicrobial resistance phenotypes was performed as described previously (9). Because the genomes exhibited <19 single-nucleotide polymorphisms (SNPs) in 4,072,405 positions (representing nearly 100% of the genomes), the isolates were suggested to be clonal.

*In silico* prediction of phage-associated sequences revealed the presence of up to two prophages. One prophage sequence is similar to that of the linear *Vibrio* satellite phage KSF-1phi (GenBank accession number AY714348) (10). The second prophage possesses sequences matching those of the phage myovirus K139 (GenBank accession number NC_003313) (11).

The genomes of the seabird isolates possess an SXT/R391-like integrative conjugative element (ICE) that is related to a 103-kb ICE*Vch*Ban8 element (GenBank accession number JQ345361) of a human pathogenic V. cholerae O37 strain (12). This ICE encodes potential virulence factors in a hot spot region of 45 kb, which might have contributed to the premature deaths of the young birds.

### Data availability.

Accession numbers for whole-genome sequences and raw sequencing reads (SRA accession numbers) are listed in Table 1.
